# Are multiple oxygen species selective in ethylene epoxidation on silver?†
†Electronic supplementary information (ESI) available. See DOI: 10.1039/c7sc04728b


**DOI:** 10.1039/c7sc04728b

**Published:** 2017-11-27

**Authors:** Emilia A. Carbonio, Tulio C. R. Rocha, Alexander Yu. Klyushin, Igor Píš, Elena Magnano, Silvia Nappini, Simone Piccinin, Axel Knop-Gericke, Robert Schlögl, Travis E. Jones

**Affiliations:** a Helmholtz-Zentrum Berlin für Materialien und Energie GmbH , BESSY II, Albert-Einstein-Straße 15 , 12489 Berlin , Germany . Email: carbonio@fhi-berli.mpg.de; b Department of Inorganic Chemistry , Fritz-Haber-Institut der Max-Planck-Gesellschaft , Faradayweg 4-6 , 14195 Berlin , Germany . Email: trjones@fhi-berli.mpg.de; c Brazilian Synchrotron Light Laboratory (LNLS) , Brazilian Center for Research on Energy and Materials (CNPEM) , PO Box 6192 , 13083-970 , Campinas , SP , Brazil; d IOM-CNR , Laboratorio TASC , S.S. 14-km 163.5 , Trieste , 34149 Basovizza , Italy; e Elettra-Sincrotrone Trieste S.C.p.A. , S.S. 14-Km 163.5 , Trieste , 34149 Basovizza , Italy; f Department of Physics , University of Johannesburg , PO Box 524, Auckland Park, 2006 , Johannesburg , South Africa; g CNR-IOM DEMOCRITOS , Consiglio Nazionale delle Ricerche—Istituto Officina dei Materiali , c/o SISSA, Via Bonomea 265 , 34136 Trieste , Italy; h Department of Heterogeneous Reactions , Max Planck Institute for Chemical Energy Conversion , Mülheim an der Ruhr 45470 , Germany

## Abstract

The nature of the oxygen species active in ethylene epoxidation is a long-standing question. While the structure of the oxygen species that participates in total oxidation (nucleophilic oxygen) is known the atomic structure of the selective species (electrophilic oxygen) is still debated. Here, we use both *in situ* and UHV X-ray Photoelectron Spectroscopy (XPS) to study the interaction of oxygen with a silver surface. We show experimental evidence that the unreconstructed adsorbed atomic oxygen (O_ads_) often argued to be active in epoxidation has a binding energy (BE) ≤ 528 eV, showing a core-level shift to lower BE with respect to the O-reconstructions, as previously predicted by DFT. Thus, contrary to the frequent assignment, adsorbed atomic oxygen cannot account for the electrophilic oxygen species with an O 1s BE of 530–531 eV, thought to be the active species in ethylene epoxidation. Moreover, we show that O_ads_ is present at very low O-coverages during *in situ* XPS measurements and that it can be obtained at slightly higher coverages in UHV at low temperature. DFT calculations support that only low coverages of O_ads_ are stable. The highly reactive species is titrated by background gases even at low temperature in UHV conditions. Our findings suggest that at least two different species could participate in the partial oxidation of ethylene on silver.

## Abstract 2

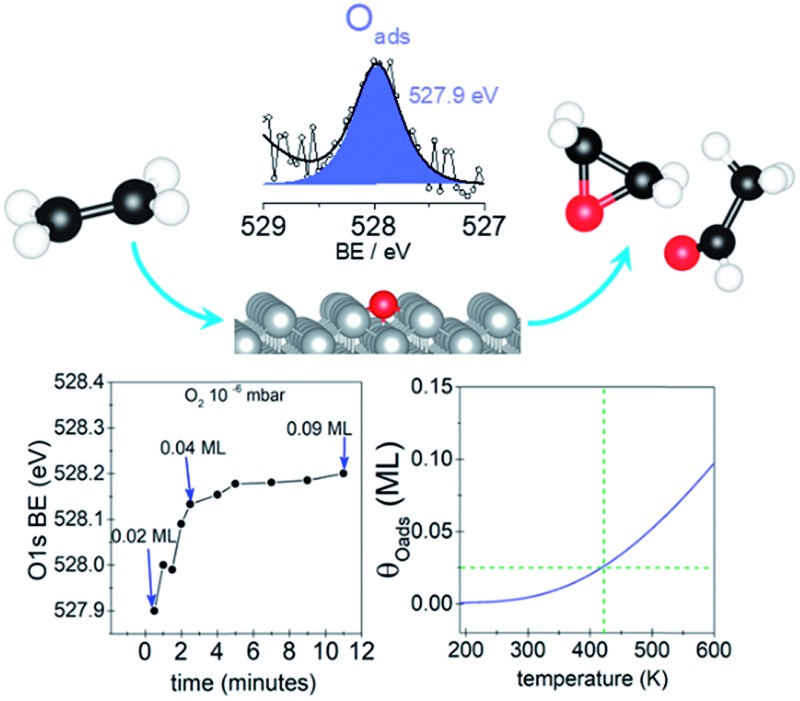
We show atomic oxygen on an unreconstructed Ag(110) surface has a O 1s binding energy ≤ 528 eV and its stable at low coverages. Our findings point to the idea of multiple selective oxygen species in ethylene epoxidation on Ag.

## Introduction

Although extensively studied,^1^–^4^ the structure of the O/Ag system during ethylene epoxidation is still not completely understood. Under reaction conditions, two broad types of oxygen species are present at the O/Ag surface during epoxidation.^5^–^7^ These species can be distinguished by X-ray Photoelectron Spectroscopy (XPS). One type (nucleophilic oxygen) has an O 1s binding energy (BE) of 528–528.5 eV and the other type (electrophilic oxygen) has a BE of 530–531 eV.^5^–^7^ The former, an electron rich oxygen, has been proposed to activate C–H bond breaking^8^,^9^ and has been shown to participate in the complete oxidation of ethylene.^10^ The latter, an electron deficient oxygen, has been proposed to open the C=C double bond of ethylene, forming the COC ring through O insertion,^9^,^11^,^12^ and has been shown to participate in epoxidation.^13^ Yet, while the atomic structure of nucleophilic oxygen is known,^10^,^14^,^15^ the atomic structure of the active species for ethylene epoxidation is debated.^4^,^6^,^15^–^17^ This electrophilic species is thought to be weakly bound oxygen and, following early assignments,^18^,^19^ has been extensively interpreted as being unreconstructed adsorbed or dissolved atomic O.^6^,^7^,^20^–^27^ However, even in early publications such assignments appeared inconsistent. Campbell *et al.*,^28^ using TPR (Temperature Programmed Reaction) and XPS, reported on an unreactive subsurface form of oxygen with BE of ∼528.5 eV and proposed that it would be similar in nature to atomic O. Similarly, in two later publications Bukhtiyarov *et al.*^29^,^30^ observed that XPS spectra would only show a main component at 528.4 eV, although the presence of dissolved O could be detected by TPD (Temperature Programmed Desorption). Consistent with the assignment of electrophilic oxygen to unreconstructed atomic oxygen, an oxometallacycle (OMC) mechanism has been developed wherein ethylene reacts with adsorbed O on the unreconstructed surface to make ethylene oxide and acetaldehyde.^17^,^31^–^33^

More recent publications from Rocca *et al.*^34^,^35^ report on O species with BE < 528 eV for a Ag(210) surface exposed to O_2_ at low temperatures. The authors attribute the low BE O 1s component to two O species: adsorbed O atoms in 4-fold hollows and O–Ag rows at the steps. Moreover, it was recently demonstrated by means of Density Functional Theory (DFT) calculations combined with experimentally measured O 1s BE and NEXAFS (Near Edge X-ray Absorption Fine Structure), that the spectroscopic features of unreconstructed atomic oxygen do not agree with those measured for the electrophilic species with a BE of 530–531 eV.^15^ Furthermore, it was shown by DFT that when Ag/O bonding is ionic, as is the case for atomic O on Ag, more weakly bound atomic oxygen will have a lower O 1s BE and thus, it cannot account for the electrophilic species.^16^ Despite this, adsorbed atomic O is still assigned in the literature to unreconstructed atomic oxygen based on the BE of 530–531 eV.^26^

On Ag(111), Carlisle *et al.*^36^ reported that unreconstructed atomic oxygen could be present only at oxygen coverages below 0.05 ML, as seen by STM. In line with this observation, an investigation^15^ with fast *in situ* XPS measurements performed with a Ag(111) single crystal in O_2_ at 10^–4^ mbar showed that the O 1s spectra had a peak with BE < 528 eV that could only be seen for the first minutes of dosing, shifting with time to slightly higher BE. The low BE component was only observed for an O-coverage of ∼0.05 ML. Although the shift in BE is small for this surface, it was interpreted as a transition from atomic oxygen on the unreconstructed silver surface to the formation of islands of O-reconstructions as coverage increased, since the reconstruction is thermodynamically favoured.^15^ In contrast, the computed BE difference of unreconstructed adsorbed atomic oxygen with respect to the O-reconstructions on the Ag(110) surface is larger.^15^ However, in the same study, for the Ag(110) surface no such low BE peak was observed when exposed to 10^–4^ mbar of O_2_. The sticking coefficient for the dissociative adsorption of O_2_ on Ag(110) has been reported to be two to three orders of magnitude higher than on Ag(111).^28^,^37^–^42^ Thus, it can be expected that the O-coverage would increase faster on Ag(110) than on Ag(111), implying a reconstructed surface would form faster on Ag(110) under O_2_.

It is well known that when a Ag(110) surface is exposed to O_2_ at conditions such that dissociative adsorption occurs, a series of *p*(*N* × 1) added row reconstructions can easily be produced.^28^,^42^ Moreover, due to the relatively high sticking coefficient of oxygen on the Ag(110) surface, these reconstructions can be made under low O_2_ dosing in UHV compatible system.^28^,^42^ Such conditions are necessary when studying highly reactive species, which can be undetectable if clean-off reactions with background gases, for instance CO, become relevant. These types of unwanted reactions are typical for adsorbed atomic O, as shown by low temperature STM measurements.^43^

Herein, we perform *in situ* experiments on a Ag(110) surface by exposing the single crystal to O_2_ at 10^–5^ mbar and 10^–6^ mbar at 423 K and taking fast O 1s spectra (30 s per spectra). We show that a low BE species is only present at low coverages (*θ*_O_ < 0.04 ML) which for Ag(110), can only be obtained at low O_2_ pressure (10^–6^ mbar). We assign this species with BE ≤ 528 eV to unreconstructed atomic O, as predicted by DFT and in line with previous interpretations.^15^ We confirm the assignment to unreconstructed atomic oxygen by XPS measurements of O/Ag(110) at 120 K in UHV where the O-reconstruction, although thermodynamically favoured,^15^ is kinetically hindered, and higher coverages (∼0.1 ML) of atomic adsorbed O on the unreconstructed silver can be obtained.

## Methodology

### Experimental details

The *in situ* XPS measurements were performed at the ISISS beamline in the BESSY II synchrotron radiation facility of the Helmholtz-Zentrum Berlin. Details about the system can be found elsewhere.^44^,^45^ The Ag(110) single crystal was polished and oriented to an accuracy <1°. The crystal was cleaned by repeating cycles of O_2_ treatment at 10^–3^ mbar at 423 K for 20 min, followed by Ar sputtering at 1.5 kV for 20 min and annealing at 673 K in vacuum (5 × 10^–8^ mbar) for 5 min. Large amounts of C, Cl, S and Si segregated to the surface after the initial O_2_ exposure, but after several cleaning cycles, only Ag and O were observed for exposures shorter than 1–2 h. The crystal was placed in a sapphire sample holder and held by a tungsten wire. Heating was done from the backside with an IR laser on a stainless-steel plate in contact with the crystal. The sample temperature was measured with a K-type thermocouple and controlled by adjusting the laser power using a PID feedback loop. Photon energies were chosen so that photoelectrons with the same kinetic energy of 150 eV could be measured for the different elements, giving an equivalent to an inelastic mean free path of 0.5 nm.

The low temperature measurements were performed in UHV at the BACH beamline in Elettra Sincrotrone Trieste.^46^,^47^ For these measurements the Ag(110) single crystal was cleaned by cycles of Ar sputtering at 1.5 kV for 20 min, annealing in O_2_ at 10^–6^ mbar at 453 K and subsequent annealing in UHV at 673 K. The cycles were repeated until no C was observed on the surface. A photon energy of 670 eV was used for acquisition of all the core-lines. For the low temperature measurements, the sample was cooled by flowing liquid N_2_ through the manipulator. Sample heating was done by a tungsten filament placed behind the sample holder. The sample temperature was measured with a N-type thermocouple. Oxygen dosing was done by backfilling the UHV chamber with O_2_ at pressures in the range 10^–7^ to 10^–6^ mbar.

In all cases, the binding energy scale for each spectra was calibrated by the Fermi edge measured with the same photon energy.

More details about the fitting procedure and other information are given in the ESI.†

### Computational details

Calculations were performed with the Quantum ESPRESSO package^48^ using the Perdew, Burke, and Ernzerhof (PBE) exchange and correlation potential^49^ and the exchange-hole dipole moment (XDM)^50^,^51^ dispersion correction. Projector augmented wave (PAW) potentials were taken from the PS library,^52^ and we employed a kinetic energy (charge density) cutoff of 70 Ry (700 Ry). Surfaces were modeled as five-layer slabs separated by 15 Å of vacuum with the bottom two layers were held fixed at their computed bulk values. A *k*-point mesh equivalent to at least (12 × 12) for the (1 × 1) surface-unit-cell was used with Marzari–Vanderbilt cold smearing^53^ and a smearing parameter of 0.01 Ry. Core level shifts were computed with the ΔSCF (Self Consistent Field) method to capture both initial and final state effects.^54^ Climbing image nudged elastic band (NEB) calculations were performed using 8–10 images with a single climbing image, a *k*-point mesh equivalent to (8 × 8) for the (1 × 1) surface-unit-cell, and a kinetic energy (charge density) cutoff of 30 Ry (300 Ry), unless otherwise noted. This reduced convergence criteria results in adsorption energy changes of only 0.1 eV.

## Results and discussion

### *In Situ* measurements

**Fig. 1:**
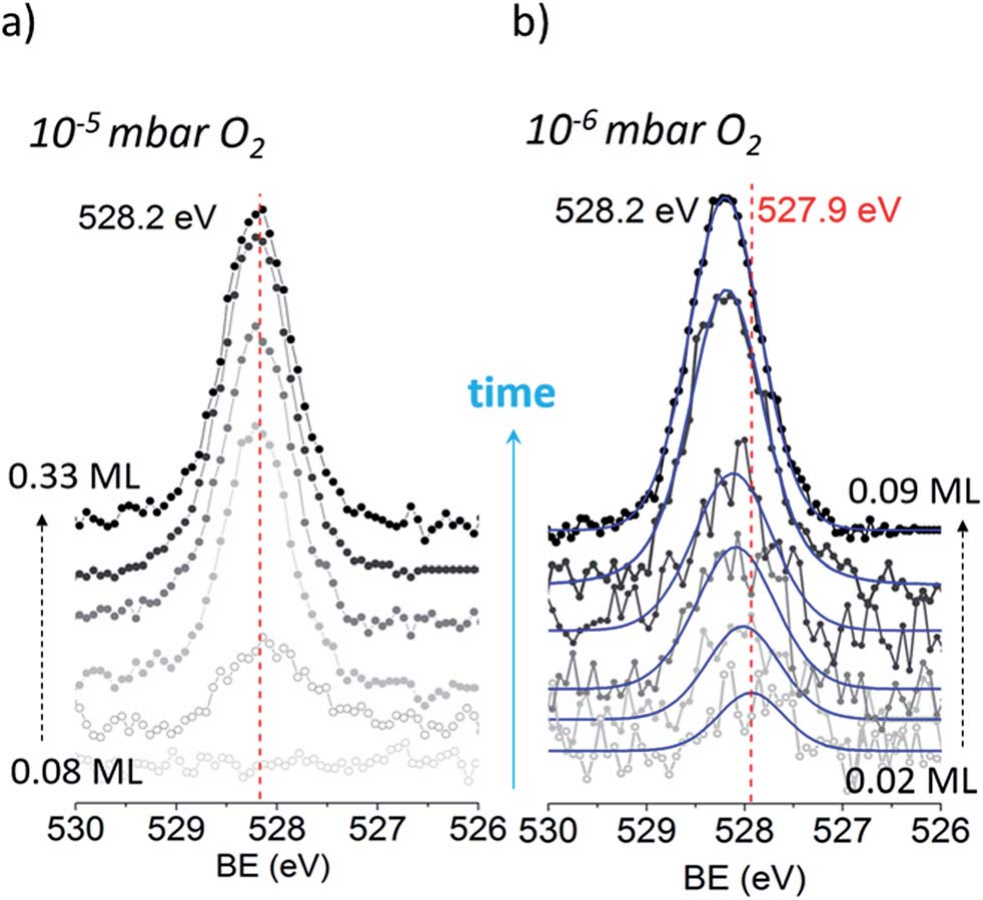
O 1s time evolution on Ag(110) during exposure to O_2_ at 10^–5^ mbar and 10^–6^ mbar at 423 K.

**Fig. 2:**
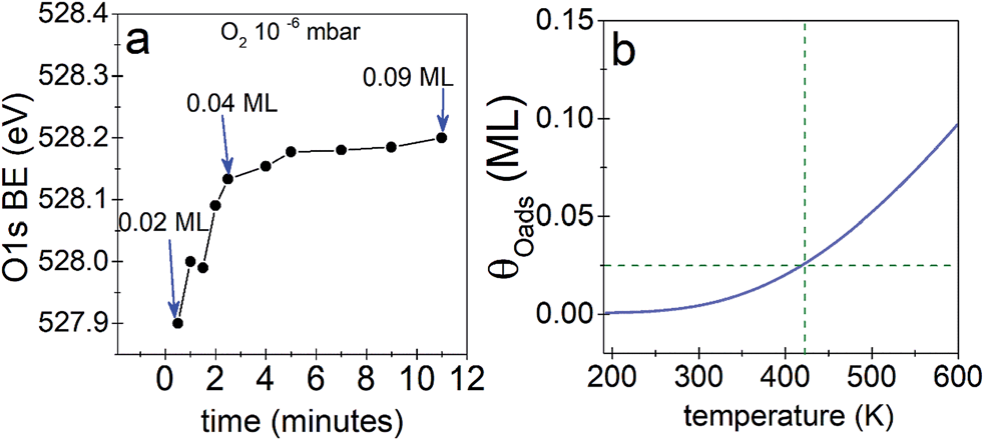
(a) O 1s BE as a function of time for Ag(110) in 10^–6^ mbar O_2_ at 423 K, (b) predicted maximum coverage of unreconstructed atomic oxygen as a function of temperature, assuming the added-row Ag–O chains in the *p*(*N* × 1) contribute no configurational entropy. The dashed line shows the predicted coverage at the temperature of the experiment.

We begin our investigation studying the interaction of O_2_ with a Ag(110) surface by performing fast *in situ* XPS measurements at low pressures, in order to characterize the species present at low coverages. When exposed to 10^–5^ mbar O_2_ at 423 K, the O 1s spectra shows only a main peak at 528.2 eV and does not change significantly with time (see Fig. 1a). This BE indicates that an O-reconstruction is formed^15^,^28^ immediately after exposure. The situation changes when the experiment is done at 10^–6^ mbar, which is close to the predicted minimum pressure needed to stabilize the *p*(*N* × 1) reconstructions at 423 K.^15^ Thus, we might expect unreconstructed atomic oxygen to be stable, at least transiently. We find that at 10^–6^ mbar O_2_ at 423 K, an oxygen species with a BE of 527.9 eV is present after 30 seconds of dosing (Fig. 1b), with an estimated coverage of ∼0.02 ML. This BE is in line with that expected for unreconstructed atomic O from DFT calculations.^15^,^16^ The O 1s BE shifts rapidly to higher BEs within the first three minutes (see Fig. 2a) reaching a BE of ∼528.15 eV and a coverage of ∼0.04 ML, indicating a surface reconstruction has precipitated at this coverage. After this point, coverage increases at a slower rate from ∼0.04 ML to ∼0.09 ML in 8 minutes and the BE of the O 1s spectra is approximately constant, reaching a value of 528.2 eV, which corresponds to that of the *p*(*N* × 1) reconstructions.^15^,^28^ This behavior is similar to what was previously observed on Ag(111) at 10^–4^ mbar^15^ and is attributed to the transition from O adsorbed on the unreconstructed silver to the formation of reconstructed *p*(*N* × 1) islands. These findings are supported by *ab initio* atomistic thermodynamics.^15^,^55^

Previous work has shown that the adsorption energy of oxygen is higher in a *p*(*N* × 1) reconstruction than on the unreconstructed surface.^15^ Inspection of Tables 1S and 2S in ESI† confirm this finding even for a low coverage of oxygen on the unreconstructed surface. Thus—ignoring changes in the vibrational free energy, which are expected to be small^55^—unreconstructed atomic oxygen will only be favored over the oxygen induced *p*(*N* × 1) reconstructions when the configurational entropy difference between the two phases is high. The difference in surface free energy for equal coverages of reconstructed and unreconstructed can then be given by:

1 Δ*γ*(*T*) = Δ*E*_ads_ – *T*Δ*S*_conf_

where Δ*E*_ads_ is the difference in adsorption energy between oxygen on the unreconstructed Ag(110) and a *p*(N × 1) reconstruction:

2 Δ*E*_ads_ = (*E*_ads,O_ – *E*_*p*(*N*×1)_)

The difference in configurational entropy in eqn (1) is:

3 Δ*S*_conf_ = *S*_conf,O_ – *S*_conf,*p*(*N*×1)_

where the configurational entropy for an adatom at coverage *θ* on the unreconstructed surface is given by:
4

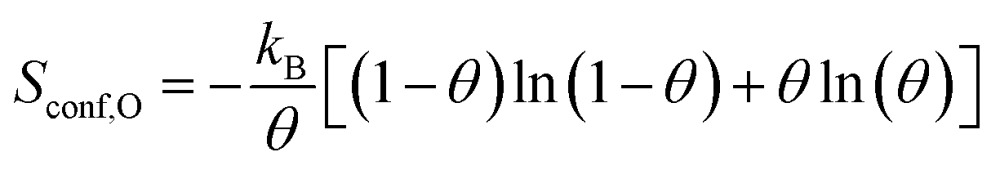




For simplicity, the configurational entropy of an O atom in the surface reconstruction is taken to be zero.

Because we are concerned with low-coverage phases, we take the adsorption energies of oxygen in their low-coverage limits, the *p*(4 × 1) reconstruction and 1/16 ML oxygen in the fourfold hollow sites of the Ag(110) surface (see ESI for more details, Table 3S and Fig. 1S†). With these adsorption energies, the maximum coverage of adsorbed oxygen on the unreconstructed surface can be found by setting Δ*γ*(*T*) = 0.

The predicted maximum coverage of oxygen on the unreconstructed surface is shown in Fig. 2b. This approach predicts a maximum coverage of atomic oxygen on unreconstructed Ag(110) of ∼0.02 to 0.03 ML at 423 K, in good agreement with the experimental results.

**Fig. 3:**
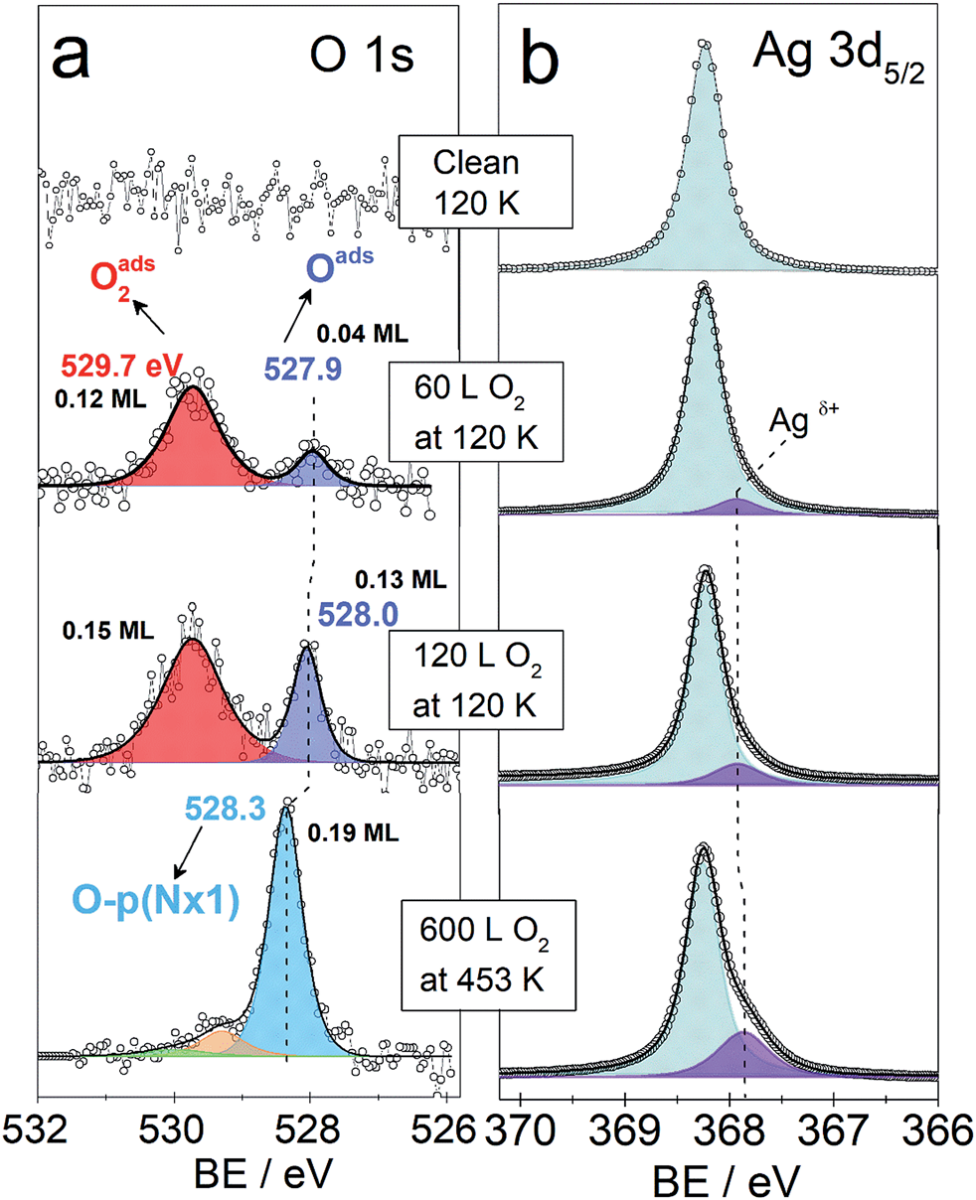
O 1s and Ag 3d_5/2_ spectra of clean and oxygen covered Ag(110) surface. Temperature and oxygen dosing are indicated in the figure.

With the preceding analysis in mind it is worth returning to the experiments performed at 10^–5^ mbar on the Ag(110). The absence of the low BE feature in these experiments can now be understood as due to the faster increase in coverage at this pressure (see Fig. 3S†). The first measured spectra after exposure of O_2_ at 10^–5^ mbar corresponds to an estimated coverage of ∼0.08 ML, a coverage at which both our experimental and theoretical (see Fig. 2) results show unreconstructed atomic O is no longer stable.

By this, it may be argued that for the pressure used in ref. 15 (10^–4^ mbar O_2_) the absence of a low BE on the Ag(110) surface is probably due to a O-coverage higher than 0.04 ML already at very short times.

It is well known that on clean silver surfaces steps act as a source of mobile Ag atoms even at room temperature^56^,^57^ and the detachment of these Ag atoms is a thermally activated process.^56^ Ostwald ripening of two dimensional islands on Ag(110) has been observed at temperatures above 160 K.^56^,^58^ The growth rate of O-reconstructions on Ag(110) at 190 K was found to be lower than the supply rate of oxygen atoms, remaining constant while the O-coverage increased.^56^ The equilibrium Ag adatom density might not be sustained at low temperatures if the Ag atom detachment from the steps is kinetically limited, even if thermodynamically favoured. Consequently, at lower temperatures unreconstructed atomic O can be expected to be present at the silver surface. Thus, we turn to low temperature experiments with the aim of obtaining unreconstructed atomic O, since this should be a stable species for longer time at low temperature and in UHV conditions.

### Low temperature measurements

We continue by analysing the interaction of O_2_ with a Ag(110) surface by dosing O_2_ at 453 K and then at 120 K. Although at 120 K the main adsorption form of oxygen is molecular, dissociation is expected due to low activation energy, which we computed to be only 0.4 eV for O_2_ dissociation along the [001] or [1–10] direction at 1/16 ML O_2_ coverage (O_2_ adsorption geometries given in Fig. 2S†). In fact, dissociation has been observed to occur to some extent already at this temperature by STM,^36^,^59^ though the formation of the added row reconstructions was found to start at 170–200 K.^43^,^59^,^60^

Taking into consideration the results obtained for the *in situ* measurements, and that the sticking coefficient for O_2_ is higher at lower temperatures,^38^–^40^,^42^ the O_2_ exposure was done using pressures in the range 10^–7^ to 10^–6^ mbar, in order to obtain low O-coverages. Moreover, low O_2_ exposures are preferred to minimize the formation of OH and CO_3_ (ref. 28 and 61) that might occur due to the fast reaction of unreconstructed atomic O with background gases CO and H_2_O.^43^

Fig. 3 shows the O 1s and Ag 3d spectra of a Ag(110) surface exposed to different amounts of O_2_ at different temperatures (indicated in the figure). The spectra acquired for the clean surface is also shown for comparison. The resulting binding energies are summarized in Table 4S.† The clean surface shows a single component with a BE of 368.25 eV for the Ag 3d_5/2_ core-line. When 600 L O_2_ is dosed at 453 K, the O 1s spectra shows a main peak at 528.3 eV. This BE is in agreement with the measured^15^,^28^ and calculated^15^ BE for the added row reconstructions on Ag(110) and in line with the measured BE from our *in situ* experiments (528.2 eV, see Fig. 1). Other small contributions are observed at 529.2 eV and 530 eV. The BEs of these species have previously been assigned to oxide-like layers and electrophilic oxygen, respectively.^6^,^15^ The corresponding Ag 3d spectra shows a main component with a BE of 368.25 eV and a smaller component at a BE of 367.85 eV, which is due to the Ag^*δ*+^ formation due to the presence of oxygen atoms on the surface.^6^,^23^ Although most metals show a core-level shift (CLS) to higher BE for higher oxidation states, it is well known that for silver there is a CLS to lower BE.^62^ The O 1s spectra for the Ag(110) exposed to 60 L and 120 L at 120 K shows two components. One at 529.7 eV was assigned to molecularly adsorbed oxygen based on our DFT calculations showing O_2_ adsorbed in a fourfold hollow (FFH) site with the interatomic axis parallel to the [001] or [1–10] direction (adsorption geometries in Fig. 2S†) has a computed O 1s BE of 529.7 eV, consistent with literature values.^28^ A second oxygen species at 527.9–528 eV is consistent with the computed O 1s binding energy of unreconstructed atomic oxygen^15^ and the *in situ* measurements of this species (see Fig. 1). Additionally, the corresponding Ag 3d spectra also show a small component at lower BE, 367.9 eV, due to Ag^*δ*+^ sites.

**Fig. 4:**
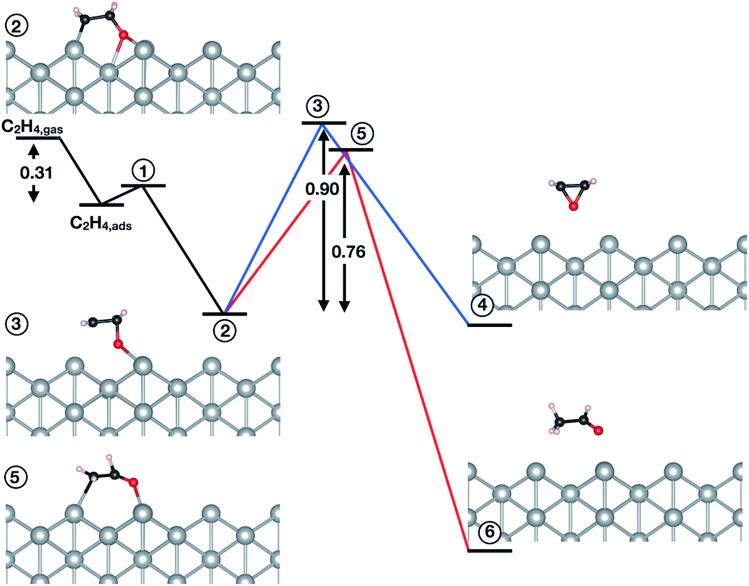
Reaction of ethylene with O_ads_ on Ag(110) in a (4 × 4) cell. The black lines indicate the formation of the OMC (step 2). The blue (red) lines show the activation energy associated with EO (AcH) formation through the transition state labeled state 3 (5).

We observe that the amount of the low BE species in the O 1s core-level spectra decreases with time. For instance, after 35 minutes at 120 K with a maximum base pressure of 10^–9^ mbar, the amount decreases by at least half (see Fig. 4S†). This is consistent with the previously reported high reactivity of unreconstructed atomic oxygen.^59^,^60^ Two kinetically different atomic O species have been previously described for CO oxidation to CO_2_.^40^,^63^,^64^ For O pre-covered Ag(110) exposed to CO, the formation of CO_2_ was observed to be slow at high oxygen coverages and to accelerate at lower coverages,^40^ due to the more reactive unreconstructed adsorbed O atoms present at the low coverage limit. Furthermore, unreconstructed adsorbed O has been reported to react with CO at temperatures as low as 70 K.^43^ It was observed by low temperature STM measurements, that the amount of adsorbed O atoms on the unreconstructed silver would decrease due to clean-off reactions with the background gas of the UHV chamber even at a base pressures lower than 1 × 10^–10^ mbar at 110 K.^43^

As mentioned before, DFT calculations predict unreconstructed atomic oxygen on silver to have a BE ≤ 528 eV (ref. 15 and 16) in contrast to the *p*(*N* × 1) reconstructions on Ag(110) for which it predicts a BE of 528.5 eV.^15^ This gives a CLS of ∼0.5 eV to lower BE for O on the unreconstructed Ag(110) surface. Thus, the BE predicted by DFT, the measured BE of 527.9–528 eV and the high reactivity observed for this species, are all characteristics consistent with atomic O adsorbed on unreconstructed Ag(110). This is consistent with the low BE component seen in the *in situ* experiments (see Fig. 1). The higher coverage attainable at low temperatures is attributed to the reconstructions' formation being kinetically hindered at 120 K,^59^,^60^ due to the lower mobility of Ag atoms at low temperature.^56^

### Adsorbed atomic O in ethylene epoxidation

The generally accepted idea that reconstructed atomic oxygen, assigned to nucleophilic oxygen, participates in the complete oxidation of ethylene to CO_2_ and that unreconstructed adsorbed atomic oxygen, often assigned to electrophilic oxygen, can participate in ethylene oxide (EO) production, motivated a series of theoretical investigations on the reaction mechanism for ethylene epoxidation.^17^,^31^–^33^ On unpromoted silver, experimental evidence^10^,^14^,^27^ shows that the reaction of ethylene with O-reconstructions favors total combustion giving CO_2_ as a product, which is supported by theory.^10^,^32^ For the reconstructions, DFT calculations show that the reaction of ethylene and atomic O has a low barrier towards acetaldehyde (AcH) formation,^10^ which rapidly burns.^10^,^14^,^31^ Conversely, for unreconstructed adsorbed atomic oxygen on silver, DFT calculations have been reported to give similar barriers for the conversion of ethylene to EO and AcH,^31^,^33^,^65^ predicting a ∼50% EO selectivity for unpromoted silver catalysts.^31^ This agrees with many experimentally observed selectivities,^1^,^2^,^66^ that were believed to take place on unpromoted silver catalysts thus, unreconstructed atomic O was thought to be the active species in ethylene epoxidation and was assigned to electrophilic oxygen.^19^

However, we demonstrate that unreconstructed atomic oxygen is present at very low O-coverages and can be only detected transiently during *in situ* measurements. At the temperature and pressure relevant for ethylene epoxidation only 2 oxygen species have been predicted to be stable by *ab initio* atomistic thermodynamic studies: the surface reconstruction (nucleophilic oxygen) and unreconstructed atomic O.^55^,^67^ The O-reconstruction is always thermodynamically favored under ethylene epoxidation conditions^15^ making unlikely that unreconstructed O would be present at the surface under equilibrium conditions. However, low coverages of such species might be present^55^,^67^ if the formation of the reconstruction was limited by kinetics, as suggested by microkinetic modeling.^68^

Now consider that on silver under O_2_ at 423–520 K nucleophilic oxygen is the first O species detected while electrophilic oxygen is obtained only after longer times of exposures.^6^,^18^ Moreover, electrophilic oxygen is more rapidly obtained by increasing the O_2_ pressure and substrate temperature^6^,^29^ or by exposing the silver catalyst to the reaction mixture (C_2_H_4_ + O_2_).^13^,^27^ Thus, adsorbed atomic oxygen on the unreconstructed silver surface which is (as shown herein) a highly reactive species detected only at low coverages its unlikely to survive under such conditions used to obtain high concentration of electrophilic oxygen for UHV studies.^13^,^27^,^29^ Furthermore, the maximum obtainable coverage of unreconstructed atomic O of *ca.* 0.02 ML (see Fig. 1 and 2) is much lower than to the O-reconstruction coverage of 0.5–0.7 ML observed in UHV^14^,^28^,^39^ and under reaction conditions.^6^,^15^ These combined characteristics of high reactivity and very low coverage have prevented the species from being accurately characterized in the past by spectroscopic techniques both in UHV and during *in situ* measurements. Considering that there is small spectroscopic difference between these species it is reasonable to think that a very low coverage of unreconstructed O atoms would remain “undetected” even by *in situ* techniques. Thus, although predicted to exist, unreconstructed atomic O has been erroneously assigned to the so called electrophilic oxygen with a BE of 530–531 eV observed on silver catalysts in UHV^13^,^27^,^29^ and under reaction conditions^5^,^7^,^20^,^21^ and shown to participate in epoxidation.^13^

The question then remains if the oxygen species identified in this work is possibly also active in epoxidation. To answer this we turned to testing the mechanism of the reaction of oxygen on the unreconstructed Ag(110) with ethylene. Following the convergence tests (see Table 5S†), we employed a (4 × 4) cell with a (3 × 3) *k*-point mesh, a kinetic energy (charge density) cutoff of 40 Ry (400 Ry), and XDM dispersion corrections. The results are summarized in Fig. 4 (see also Table 6S†). Inspection of Fig. 4 reveals ethylene can react with a low coverage (*θ* = 1/16 ML) of oxygen adsorbed on the unreconstructed Ag(110) through an oxometallacycle (OMC) mechanism, as has been observed for other surfaces^31^,^66^,^69^,^70^ and a high coverage (*θ* = 1/4 ML) of oxygen on the unreconstructed Ag(110).^31^ The first step in the reaction is ethylene adsorption, which is 0.31 eV exothermic when dispersion corrections are included. Following adsorption, the (partial) oxidation of ethylene proceeds by OMC formation, as the C–H bond is too strong to make hydrogen abstraction from ethylene feasible.^31^ We find that OMC formation is weakly activated, with *E*_a_ = 0.09 eV.

Both EO and AcH can be formed through decomposition of the surface OMC. Assuming the EO produced in this reaction does not further decompose—isotope labeling studies on silver sponge suggest 10% of the EO is burned^71^—and noting that AcH rapidly combusts^72^,^73^ allows the branching ratio associated with EO and AcH formation to be viewed as a computational measure of the maximum selectivity afforded by an OMC mechanism.^10^,^31^,^66^,^69^,^74^–^76^ From this measure one would predict the low-coverage phase of O_ads_ investigated in this work is not selective to EO, with the barrier to AcH formation (0.76 eV) 0.14 eV lower than the barrier to EO formation (0.90 eV).

It is interesting to note, however, that at a higher coverage the OMC is thought to decompose more selectively towards EO.^31^,^77^ To test this we recomputed the branching ratio using a (2 × 2) cell and found no evidence increasing the OMC coverage will change the preference to AcH (see Table 7S and 8S†). We further verified the absence of dispersion corrections and a different pseudopotential library do not appreciably alter the computed branching ratio (see Table 7S†).

Our results then suggest the oxygen adsorbed on unreconstructed Ag(110) identified in this work will not be selective towards EO. However, the small difference in AcH/EO activation energy implies O_ads_ may produce EO as a minority product with AcH, though perhaps less so than oxygen on unreconstructed Ag(111) or Ag(100).^31^,^66^,^69^ This behavior is in contrast to reconstructed atomic oxygen on the Ag(110) which—with an *E*_a_ to AcH more than 0.4 eV lower than that to EO^10^—is selective towards AcH, and hence, CO_2_. Furthermore, in the presence of promoters the AcH/EO branching ratio associated with the reaction of O_ads_ on Ag(110) may shift towards EO. Such behavior has already been found in calculations of the OMC mechanism in the presence of halogens^74^ and Cs^76^,^78^ on Ag(111).

The combination of the experimental evidence show that unreconstructed O and electrophilic oxygen are different species and DFT indicates that O on the unreconstructed surface may participate in the partial oxidation of ethylene. Thus, two species may be active in epoxidation. First, the covalently bond type of oxygen^16^ (of debated structure) with a BE of 530–531 eV (electrophilic oxygen).^5^,^6^,^13^,^29^,^79^ Second, the O adsorbed on the unreconstructed surface with a BE ≤ 528 eV as shown herein. The ultimate test for the later will be the experimental epoxidation of ethylene with a low coverage of O adsorbed on an unreconstructed surface. While DFT calculations have shown that the reaction of ethylene with O_ads_ can produce EO through an oxometallacycle (OMC) intermediate,^17^,^33^ the experimental evidence on this mechanism has relied on the production of EO from an OMC formed after EO adsorption on a silver single crystal.^77^ Here we have shown that O_ads_ can be prepared and identified in UHV, opening the opportunity to test the complete route of reaction C_2_H_4_ + O_ads_ → OMC → EO predicted by DFT, although the high reactivity of O_ads_ towards clean-off reactions will make such studies challenging.

## Conclusions

We have determined experimentally that on unreconstructed Ag(110) adsorbed atomic O has a BE ≤ 528 eV, as earlier predicted by DFT, which is lower than the *p*(*N* × 1) reconstruction and thus, cannot give rise to the O 1s feature with BE of 530–531 eV (electrophilic oxygen) believed to be responsible for epoxidation.

Atomic O adsorbed on the unreconstructed silver surface is present during *in situ* experiments upon O_2_ exposure at low O-coverages (<0.04 ML) at 423 K. At higher coverages, the thermodynamically favored O-reconstructions are formed. These findings are supported by DFT. At low temperatures, *ca.* 120 K, unreconstructed O can be obtained in UHV and the atomic O coverage reaches 0.1 ML, due to kinetic limitations to form the O-reconstructions, which is a thermally activated process. This species is highly reactive towards clean-off reactions even at 120 K and reacts rapidly with background gases. These findings suggest that only very low coverage of unreconstructed atomic O is likely to be present at the silver surface under ethylene epoxidation conditions. Although present at low coverage, the computed barriers to EO and AcH indicate that on unpromoted silver EO might be produced as a minority product through reaction with oxygen adsorbed on unreconstructed Ag(110). Our findings suggest that at least two different species, a covalently bond oxygen—electrophilic oxygen—(with a BE of 530–531 eV) and unreconstructed atomic oxygen (with a BE ≤ 528 eV) might participate in the partial oxidation of ethylene. This points to a new way of thinking about one of the most well-studied reactions in chemistry. The fact that not one but multiple oxygen species can participate in epoxidation. This has important implications for the understanding of the mechanism of ethylene epoxidation on silver and of the role of the different oxygen species.

## Conflicts of interest

There are no conflicts to declare.

## Supplementary Material

Supplementary information
