# Supplementary material for: Are multiple oxygen species selective in ethylene epoxidation on silver?
Source: Chem Sci. 2017 Nov 27;9(4):990–8. doi: 10.1039/c7sc04728b (PMC5874983; doi:10.1039/c7sc04728b)
Supplement: Supplementary file 1 [file SC-009-C7SC04728B-s001.pdf]

Electronic Supplementary Material (ESI) for Chemical Science.
This journal is © The Royal Society of Chemistry 2017

Supporting Information for

Are multiple Oxygen Species Selective in Ethylene Epoxidation on Silver?

Emilia A. Carbonio[a,b,‡], Tulio C.R. Rocha[c], Alexander Yu.Klyushin[a,b], Igor Píš[d,e], Elena Magnano[d,f], Silvia Nappini[d], Simone Piccinin[g], Axel Knop-Gericke[b], Robert Schlögl[b,h], Travis E. Jones[b†]

[a]Helmholtz-Zentrum Berlin für Materialien und Energie GmbH, BESSY II, Albert-Einstein-Straße 15, 12489 Berlin, Germany.

[b]Department of Inorganic Chemistry, Fritz-Haber-Institut der Max-Planck-Gesellschaft, Faradayweg 4-6, 14195 Berlin, Germany.

[c]Brazilian Synchrotron Light Laboratory (LNLS), Brazilian Center for Research on Energy and Materials (CNPEM), PO Box 6192, 13083-970, Campinas, SP.Brazil.

[d]IOM-CNR, Laboratorio TASC, S.S. 14-km 163.5, 34149 Basovizza, Trieste, Italy.

[e]Elettra-Sincrotrone Trieste S.C.p.A., S.S. 14-Km 163.5, 34149 Basovizza, Trieste, Italy.

[f]Department of Physics, University of Johannesburg, PO Box 524, Auckland Park, 2006, Johannesburg, South Africa.

[g]CNR-IOM DEMOCRITOS, Consiglio Nazionale delle Ricerche—Istituto Officina dei Materiali, c/o SISSA, Via Bonomea 265, 34136 Trieste, Italy.

[h]Department of Heterogeneous Reactions, Max Planck Institute for Chemical Energy Conversion, Mülheim an der Ruhr 45470, Germany.

[‡]carbonio@fhi-berli.mpg.de

[†]trjones@fhi-berli.mpg.de

Computational Details on the adsorption of O_2 and O.

The adsorption energies of atomic O on the unreconstructed silver surface and atomic O in the p(Nx1) reconstructions are defined as:

$$E_{ads,O} = E_{total,Oads} - E_{clean} - \frac{1}{2}E_{O2} \quad (1S)$$

and

$$E_{p(N\times1)} = E_{total,p(N\times1)} - E_{clean} - \frac{1}{2}E_{O2} - E_{Ag}. \quad (2S)$$

In equation (1S) $E_{total,Oads}$ is the total energy—computed with DFT—of a Ag(110) slab with a single adsorbed oxygen atom, E_{clean} is the energy of the same slab without oxygen, and E_{O2} is the energy of an O_2 molecule. The terms in equation (2S) are analogous—$E_{total,p(N\times1)}$ is the energy of a Ag(110) slab with a $p(N\times1)$ reconstruction, E_{clean} is the energy of the corresponding clean slab, and E_{Ag} is the energy of a bulk silver atom. E_{O2} was computed as:

$$E_{O2} = 2\left(E_{Ag_2O} - 2E_{Ag} - \Delta H^f_{Ag_2O}\right), \quad (3S)$$

where the experimental value of $\Delta H^f_{Ag_2O}$ extrapolated to 0 K, -0.325 eV [7], was used to avoid the well-known overbinding of O_2 in PBE DFT.

The structures needed for the energies in equations (2S) and (3S) have to be chosen. To make this choice we need to examine the low coverage limits, where the configurational entropy will be largest. These tests were performed with a kinetic energy cutoff of 70 Ry and **k**-point mesh equivalent to (12×12) for the (1×1) surface unit cell.

For the p(N×1) reconstructions we find that the adsorption energy per oxygen is nearly independent of N for N=1,2,3, see Table 3S. Thus, the p(4×1) phase was selected to reflect the low coverage limit.

Table 1S. Convergence of adsorption energy, given in eV, for the p(4×1) added row reconstruction. The **k**-point mesh is given as the equivalent for the (1×1) cell. Adsorption energy are in eV.

	Adsorption energy / eV (see equation 2S)				
**k** points	Ecut 30 Ry	Ecut 40 Ry	Ecut 50 Ry	Ecut 60 Ry	Ecut 70 Ry
12×12	0.73	0.75	0.75	0.74	0.74
16×16	---	0.75	---	0.75	0.75
20×20	---	0.78	---	0.76	0.76
24×24	---	0.77	0.78	0.76	0.76

Table 2S. Convergence of adsorption energy, given in eV, for 1/16 ML oxygen on the four fold hollow sites of the Ag(110) surface. The **k**-point mesh is given as the equivalent for the (1×1) cell. Adsorption energy are in eV.

	Adsorption energy / eV (see equation 1S)				
**k** points	Ecut 30 Ry	Ecut 40 Ry	Ecut 50 Ry	Ecut 60 Ry	Ecut 70 Ry
12x12	0.58	0.61	0.61	0.61	0.62
20x20	---	0.59	---	---	---
24x24	---	0.60	0.59	0.59	0.59

For oxygen adsorbed on the unreconstructed Ag(110) we find that $E_{ads,O}$ is sensitive to coverage. This behavior is plotted in Figure 1S for both the results with and without dispersion corrections. To facilitate plotting the two results together they are plotted as $\Delta E_{ads,O} = E_{ads,O}(\theta) - E_{ads,O}(\theta = 1/36)$, that is the change in $E_{ads,O}$ versus the lowest coverage considered. The filled (empty) circles show the results with (without) dispersion corrections. At high, 1/4 ML, oxygen coverage there is repulsion between neighboring adsorbates, as the adsorbed O is formally O^{II-}. As the coverage is reduced from 1/4 ML to 1/36 ML this repulsive interaction diminishes and $E_{ads,O}$ can be seen to converge to the low-coverage limit by ~1/16 ML. Thus, $E_{ads,O}$ at 1/16 ML coverage was used as the low-coverage limit.

Table 3S. $E_{p(N\times1)}$ for N=2-4 computed with a kinetic energy cutoff of 70 Ry and **k**-point mesh equivalent to (12×12) for the (1×1) surface unit cell. Adsorption energy are in eV.

Structure	$E_{p(N\times1)}$ / eV
p(2×1)	0.74
p(3×1)	0.75
p(4×1)	0.74

With the adsorption energies of oxygen in the low-coverage phases defined—taken from a p(4×1) reconstruction and 1/16 ML oxygen in the four fold hollow (FFH) sites of the Ag(110) surface computed with a kinetic energy cutoff of 70 Ry and **k**-point mesh equivalent to (24×24) for the (1×1) surface unit cell—the maximum coverage of adsorbed oxygen on the unreconstructed surface was computed by setting $\Delta\gamma(T) = 0$ (see Figure 3).

Figure 2S shows the structure for the adsorption of O_2 and atomic O on the unreconstructed Ag(110) and for the p(4×1) reconstruction.

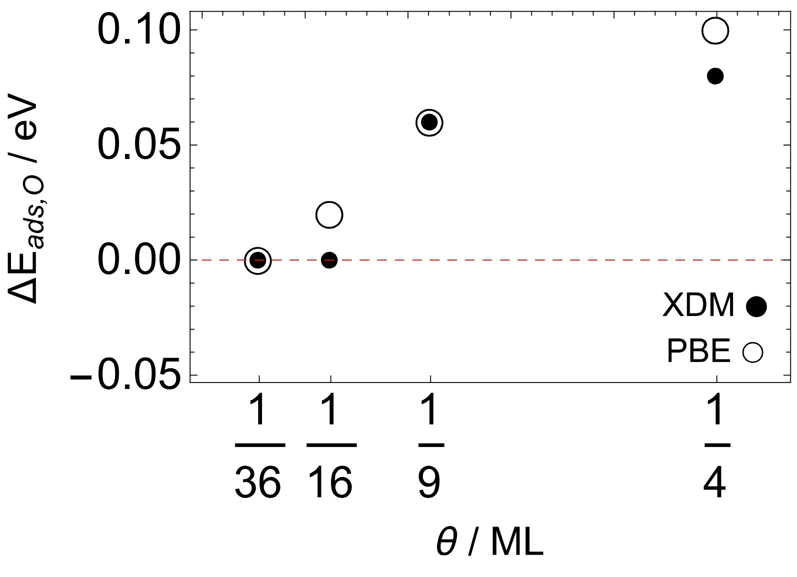

Figure 1S. Change in adsorption energy computed with a kinetic energy cutoff of 70 Ry and **k**-point mesh equivalent to (12×12) for the (1×1) surface unit cell. Adsorption energy are in eV.

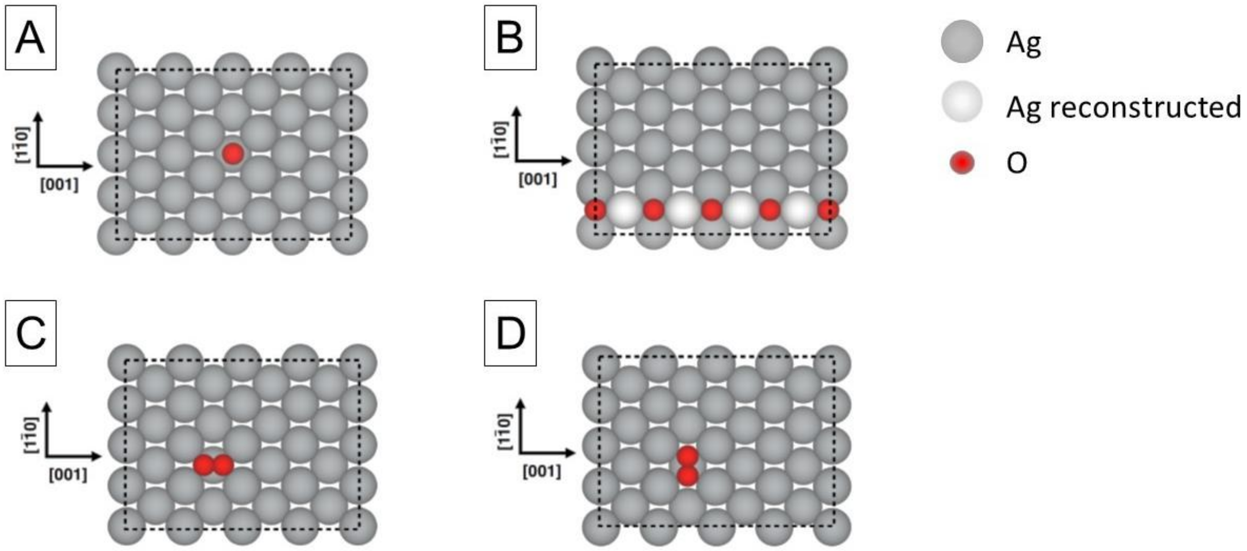

Figure 2S. (4 × 4) cell showing atomic O adsorbed on the unreconstructed Ag(110) surface (A), the p(4x1) reconstruction (B), O_2 adsorbed on Ag(110) along the (001) direction (C) and the (1-10) direction (D). O is red, Ag is grey and Ag reconstructed is white.

Experimental details of in-situ measurements.

For quantitative analysis, least-square fitting of the spectra was used. For deconvolution, a mixed Gaussian/Lorentzian (70%/30%) line shape was used for the O 1s components. For the Ag 3d a Doniach-Sunjic (DS) lineshape convoluted with a Gaussian function was used. The asymmetry parameter of the DS was fixed as 0.03[1] and the Gaussian broadening was determined for the clean silver and kept constant.

Figure 3S shows the evolution of O-coverage with time obtained from the XPS data recorded during in-situ measurements of Ag(110) exposed to O_2 at 10^{-5} mbar and 10^{-6} mbar. On Ag(110) it was observed by STM that at oxygen pressures below 10^{-5} mbar the mass transport of Ag is due to detachment and diffusion of Ag adatoms from the kinks and steps to the terraces. However, at higher O_2 pressure (P $\geq 10^{-5}$ mbar) Ag atoms are also supplied by a substrate extraction channel which provides Ag atoms also from the terraces, creating pits and changing the surface morphology[2] thus, creating more steps and defect sites on which O_2 dissociation is facilitated[3, 4], increasing the O atom supply.

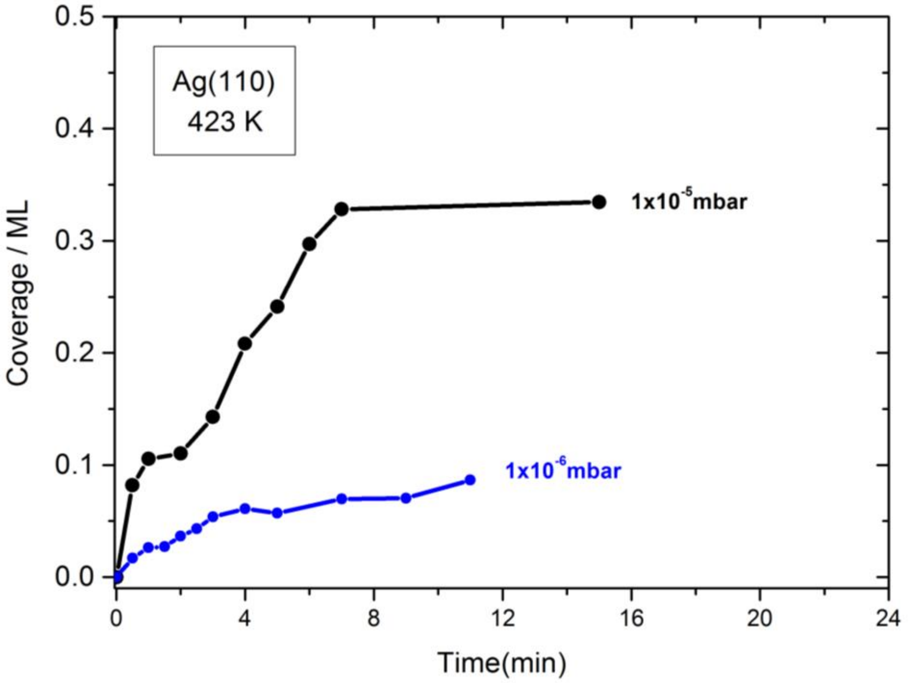

Figure 3S. Evolution of O-coverage on Ag(110) with time at 10^{-5} mbar and 10^{-6} mbar of O_2. The coverage was estimated from in-situ XPS measurements.

Experimental details of low temperature measurements.

Table 4S shows the BEs obtained for the different components (and their assignment) for the Ag 3d and O 1s spectra in the UHV experiments (for the spectra shown in Figure 3). The BE for unreconstructed atomic O (O ads) has a CLS of ~0.4 eV to lower BE with respect to the atomic O in the reconstructions (O-p(Nx1)). The CLS for $Ag^{\delta+}$ is ~0.4 eV for the O-p(Nx1) and ~0.3 eV for unreconstructed O, consistent with a weakly adsorbed O were less charge is transferred from silver to oxygen.[5]

Figure 4S shows how the low BE component of O 1s spectra decreases with time, due to the reaction with background CO and H_2O from the UHV chamber. The appearance of a higher BE component at 530.2 eV is consistent with the formation of CO_3.[1, 6]

Table 4S. Binding Energies (in eV) obtained from the deconvolution of the Ag 3d and O 1s spectra recorded for clean and O-covered Ag(110) at 453 K and 120 K, shown in Figure 4.

	Assignment	Clean Ag	60 L O_2 at 120 K	120 L O_2 at 120 K	600 L O_2 at 453 K
Ag 3d	Ag^0	368.22	368.25	368.23	368.23
	$Ag^{\delta+}$	-	367.93	367.92	367.85
O 1s	O_2 ads	-	529.72	529.74	-
	O-p(Nx1)	-	-	-	528.3
	O ads	-	527.9	528.0	-

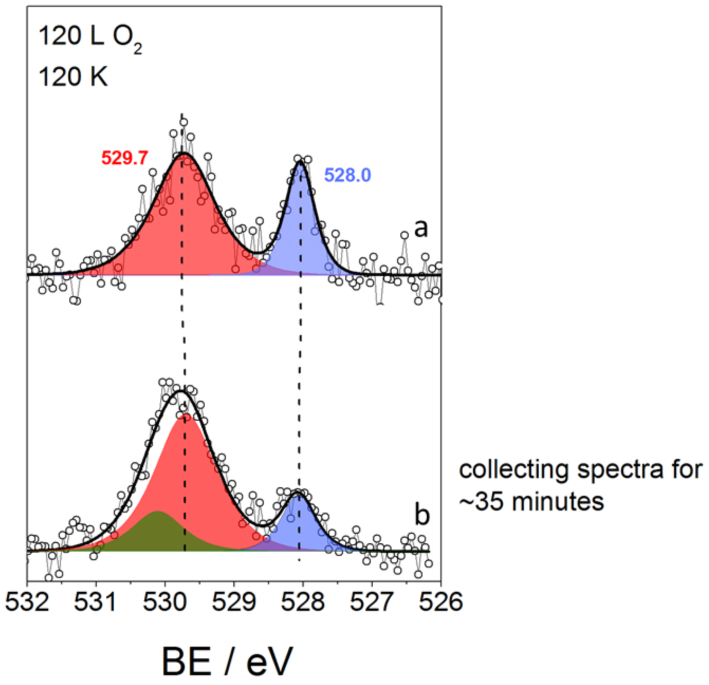

Figure 4S. O 1s spectrum measured in UHV after Ag(110) was exposed to O_2 at 120 K (a). O1s spectrum measured in UHV after 35 minutes (b).

Computational Details on the reaction of O_{ads} with C_2H_4

Table 5S: Effect of kinetic energy cutoff on computed barriers for the (4 × 4) cell. The charge density cutoff is always ten times the kinetic energy cutoff.

Ecut / Ry	Ea EO / eV	Ea AcH / eV	Ea EO XDM / eV	Ea AcH XDM / eV
30	0.93	0.78	0.91	---
40	0.94	0.80	0.90	0.77

Table 46S: Energies at stationary points along the minimum energy path for the reaction of ethylene with O_{ads} in a (4 × 4) cell computed with a (3 × 3) **k**-point mesh, a kinetic energy (charge density) cutoff of 40 Ry (400 Ry), and XDM dispersion corrections. $C_2H_{4,gas}$ and O_{ads} is set to the zero of energy. For convenience the activation energies are also listed.

Structure	Step label in Figure 4	Energy / eV	*Ea* / eV
$C_2H_{4,gas}$		0.00	---
$C_2H_{4,ads}$		-0.31	---
TS to OMC	1	-0.22	0.09
OMC	2	-0.83	---
TS to EO	3	0.07	0.90
EO_{ads}	4	-0.88	---
EO_{gas}		-0.80	---
TS to AcH	5	-0.06	0.76
AcH_{ads}	6	-1.95	---
AcH_{gas}		-1.81	---

The 0.14 eV difference in activation energy to AcH and EO implies O_{ads} will be selective to AcH. However, this difference should be treated with caution as, for instance, the reported mean signed error associated with the PBE exchange and correlation potential underlying our calculations is roughly 0.1 eV for the adsorption reaction of closed shell molecules.[8] And while the dispersion

corrections may improve this value somewhat we cannot rule out a low coverage of O_{ads} can produce EO as a minority product.

Table 7S: Activation energies computed for EO and AcH formation through the OMC mechanism at two OMC coverages with and without dispersion corrections.

Structure	Ea EO / eV	Ea AcH / eV	Ea EO XDM / eV	Ea AcH XDM / eV
(2 × 2)	0.91	0.79	0.87	0.73
(4 × 4)	0.94	0.80	0.90	0.77

Table 8S: Activation energies computed for EO and AcH formation through the OMC mechanism using PS Library PAW potentials, SSSP Δ_{acc} = 0.31 meV Library [9] potentials, and ultrasoft pseudopotentials without a non-linear core correction from the original QE library. XDM corrections were not included. For the PS library results with a 7 layer, as opposed to 5 layer, slab are also included.

Structure	Ea EO / eV Ecut 40 Ry	Ea AcH / eV Ecut 40 Ry	Ea EO / eV Ecut 50 Ry	Ea AcH / eV Ecut 50 Ry
PS	0.91	0.79	---	---
PS (7 layer)	0.92	0.82	---	---
SSSP	0.92	0.81	0.92	0.81
van	0.92	0.85	---	---

References

1. Rocha, T.C.R., et al., *The silver-oxygen system in catalysis: new insights by near ambient pressure X-ray photoelectron spectroscopy.* Physical Chemistry Chemical Physics, 2012. **14**(13): p. 4554-4564.

2. Pai, W.W., et al., *Steps as Adatom Sources for Surface-Chemistry - Oxygen Overlayer Formation on Ag(110).* Surface Science, 1995. **330**(3): p. L679-L685.

3. Albers, H., W.J.J. Van Der Wal, and G.A. Bootsma, *Ellipsometric study of oxygen adsorption and the carbon monoxide-oxygen interaction on ordered and damaged Ag(111).* Surface Science, 1977. **68**: p. 47-56.

4. Su, D.S., et al., *Surface chemistry of Ag particles: Identification of oxide species by aberration-corrected TEM and by DFT calculations.* Angewandte Chemie-International Edition, 2008. **47**(27): p. 5005-5008.

5. Jones, T.E., et al., *Insights into the Electronic Structure of the Oxygen Species Active in Alkene Epoxidation on Silver.* Acs Catalysis, 2015. **5**(10): p. 5846-5850.

6. Campbell, C.T. and M.T. Paffett, *The Interactions of O2, Co and Co2 with Ag(110).* Surface Science, 1984. **143**(2-3): p. 517-535.

7. Li, W.-X.; C. Stampfl; M. Scheffler, *Insights into the Function of Silver as an Oxidation Catalyst by ab initio Atomistic Thermodynamics.* Physical Review B, 2003, 68, 165412.

8. Duanmut, K.; Truhlar, D.G., *Validation of Density Functionals for Adsorption Energies on Transition Metal Surfaces*. J. Chem. Theory Comput.*, 2017.**13** , p. 835–842*

9. SSSP: I.E. Castelli, N. Mounet, A. Marrazzo, G. Prandini and N. Marzari, in preparation (2016) http://materialscloud.org/sssp.
